# Expression, purification of herpes simplex virus type 1 US11 Protein, and production of US11 polyclonal antibody

**DOI:** 10.1186/1743-422X-8-490

**Published:** 2011-10-31

**Authors:** Yizhong Huang, Wuyunerdeni, Shanglong Yao

**Affiliations:** 1 Department of Anesthesiology, Union Hospital, Tongji Medical College, Huazhong University of Science and Technology, 1277 Jiefang Avenue, Wuhan, 430022, China

**Keywords:** Herpes simplex virus type 1 (HSV-1), US11 protein, Protein expression, Polyclonal antibody, Immunofluorescent assay

## Abstract

**Background:**

The US11 protein of herpes simplex virus type 1 (HSV-1) is a small, highly basic phosphoprotein expressed at late times during infection. To date, the function of US11 protein in cell culture and animal models is poorly understood. To further investigate the function of the US11 protein, this study was undertaken to express the US11 protein and raise a polyclonal antibody.

**Results:**

The US11 gene was cloned into the prokaryotic expression vector pET-32a (+) to express His-tagged US11 protein in Escherichia coli. After purification by nickel affinity chromatography and refolding, the recombinant protein was used to raise the anti-US11 polyclonal antibody. Western blot analysis demonstrated that the US11 protein was specifically recognized by the polyclonal antibody, and immunofluorescent assay also showed that the antibody was able to probe the US11 protein in the cells infected with HSV-1.

**Conclusions:**

In the present study, we obtained a high-level expression of the recombinant US11 protein as well as high titers of rabbit polyclonal antibody specially against US11 protein in HSV-1 infected cells. This special polyclonal antibody provides a good tool for further studying structural and functional characterization of HSV-1 US11 protein.

## Background

Herpes simplex virus type 1 (HSV-1) is a large DNA virus that latently infects neurons and periodically reinitiates productive growth at epithelial sites, causing blisters, or in the central nervous system, resulting in encephalitis. During productive infection, the 152-kb double-stranded HSV-1 genome is rapidly translocated to the nucleus where at least 80 viral genes are transcribed by the host cell RNA polymerase II (Pol II) [1]. Expression of the viral genes occurs in a coordinately activated cascade fashion that consists of the sequential expression of immediate-early (IE), early (E), and late (L) genes [2]. The US11 protein expresses at late times during HSV-1 infection and is one of the late genes of HSV-1 [3].

The US11 protein is a 21 kDa, highly basic phosphoprotein [4], and is also an RNA-binding protein, post-transcriptional regulator of gene expression [5-7]. US11 is present in the nucleus, particularly concentrated in the nucleolus, and the cytoplasm [8,9] and is present in the virion as a component of the tegument (approximately 600 to 1,000 molecules per virion). Furthermore, US11 interacts with several different cellular proteins such as human ubiquitous kinesin heavy chain (uKHC) [10], homeodomain interacting protein kinase 2 (HIPK2) [11], double-stranded RNA-dependent protein kinase (PKR) and a dsRNA-independent protein activator of PKR (PACT) [12,13]. US11 has been reported as a potent inhibitor of PKR activation through binding to dsRNA [14] or through direct interaction with PKR in the context of viral infection [12] and therefore could interfere with the PKR mediated host cell responses. Finally, US11 has been recently shown to also counteract the activity of the 2'-5' oligoadenylate synthetase (OAS), a cellular protein critical for host cell defense [15]. Therefore, it is clear that US11 is a multifunctional protein involved in HSV-1 infection.

In the present study, the US11 gene was cloned into pET-32a(+) to yield pET-32a-US11. The His-tagged US11 protein was then expressed in *E. coli *BL21 (DE3) cells and purified by a nickel-nitrilotriacetic acid (Ni^2+^-NTA) affinity resin under denaturing conditions. Subsequently, a polyclonal antibody was raised against the purified His-tagged US11 protein in rabbits. Finally, the reactivity and specificity of the polyclonal antibody were characterized by Western blot and immunofluorescent assays.

## Results

### Construction of the US11 prokaryotic expression plasmid

**Figure 1:**
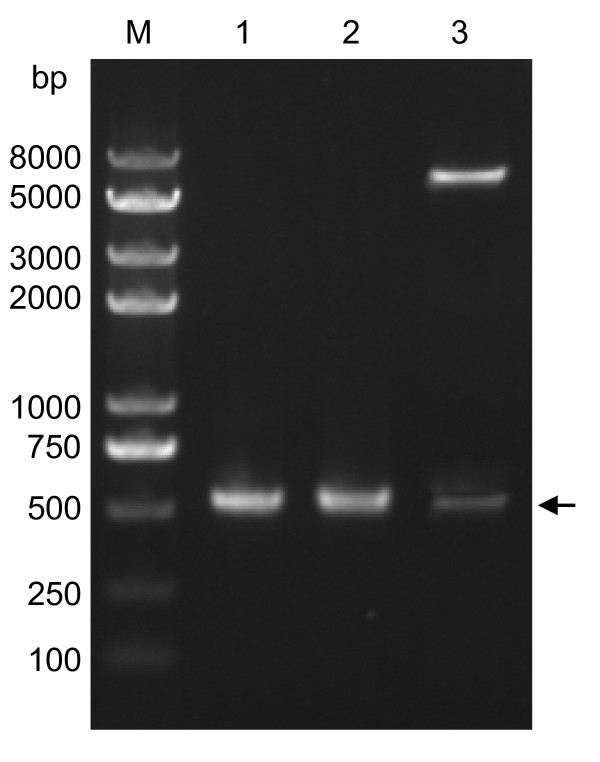
**Construction of the recombinant plasmid pET-32a-US11**. Lane 1, the PCR product of the US11 gene; Lane 2, the recombinant plasmid pET-32a-US11 was confirmed by PCR; Lane 3, the recombinant plasmid pET-32a-US11 digested with *Eco*RI and *Sal*I; and Lane M, the DNA marker. Arrowhead indicates the position of the US11 fragment.

The full-length US11 gene, which is composed of 459 bp (base pairs) and predicted to encode a protein of 152 amino acids, was amplified successfully from the HSV-1 (strain F) genome (Figure 1, lane 1). The PCR product was digested with *Eco*RI and *Sal*I and inserted into pET-32a (+) digested with the same enzymes to yield the recombinant expression plasmid pET-32a-US11. Then, the recombinant plasmid was verified by colony PCR (Figure 1, lane 2) and restriction enzymes digestion (Figure 1, lane 3). The sequencing result also showed that there was no mutation of amino acid sequences (data not shown).

### Expression of the His-tagged US11 protein

**Figure 2:**
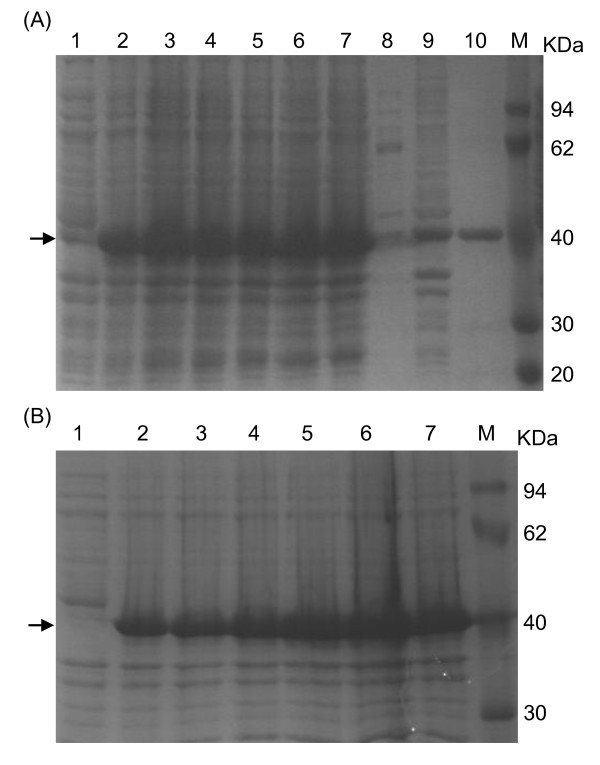
**Expression analysis and optimization of the expression for the His-tagged US11 protein**. (A) Analysis of expressed His-tagged US11 protein and optimization of the IPTG concentration and induction temperature. Lane 1, total protein from BL21 (DE3) with pET32a-US11 before induction; Lanes 2-5, total protein from pET32a-US11 transformed BL21 (DE3) after induction with the concentrations of IPTG: 0.5, 1, 1.5 and 2 mM, respectively. Lanes 6 and 7, total protein from pET-32a-US11 transformed BL21 (DE3) after induction at 30°C and 37°C, respectively. Lane 8, soluble fractions; Lane 9, insoluble fractions; Lane 10, the purified US11 protein after dialysis; Lane M, the protein maker. (B) Optimization of the induction duration. Lanes 1-7, total protein from pET32a-US11 transformed BL21 (DE3) after induction with IPTG (1 mM) for 0, 1, 2, 3, 4, 5 and 6 h, respectively, at 37°C. Lane M, the protein maker. Arrowheads indicate the position of the His-tagged US11 protein.

After induction with 1.0 mM IPTG at 37°C for 4h, *E. coli *BL21 (DE3) harboring pET-32a-US11 exhibited a high level of expression (Figure 2A, lane 3). A distinct band of approximately 40 kDa, corresponding to the expected molecular weight of the His-tagged US11 protein, was found only after induction (Figure 2A, lanes 2-7), whereas there was no expression of the US11 protein in BL21(DE3) harboring pET32a-US11 without IPTG induction (Figure 2A, lane 1).

Additionally, according to the SDS-PAGE analysis of the soluble fraction and cell debris pellet (Figure 2A, lanes 8 and 9), the majority of the induced protein was found in the cell debris pellets (Figure 2A, lane 9), suggesting that the His-tagged US11 protein was insoluble in the form of inclusion bodies. Meanwhile, several expression parameters, including IPTG concentrations (Figure 2A, lanes 2-5), induction temperatures (Figure 2A, lanes 6 and 7) and induction times (Figure 2B), were tested to optimize the expression of the US11 protein as previously described [16]. As results, the recombinant protein was found to have the highest expression under the following condition, namely 1 mM IPTG (Figure 2A, lane 3) for 5 h (Figure 2B, lane 6) at 37°C (Figure 2A, lane 7).

### Purification of the His-tagged US11 protein

The His-tagged US11 protein was purified as previously described [16]. The SDS-PAGE results verified the successful purification, because only one clear band with molecular weight about 40 kDa was observed (Figure 2A, lane 10). After purification, the His-tagged US11 protein was quantified. The result showed that the concentration of the purified His-tagged US11 protein was 911.3 μg/ml (data not shown).

### Production and characterization of the polyclonal antibody against purified His-tagged US11 protein

**Figure 3:**
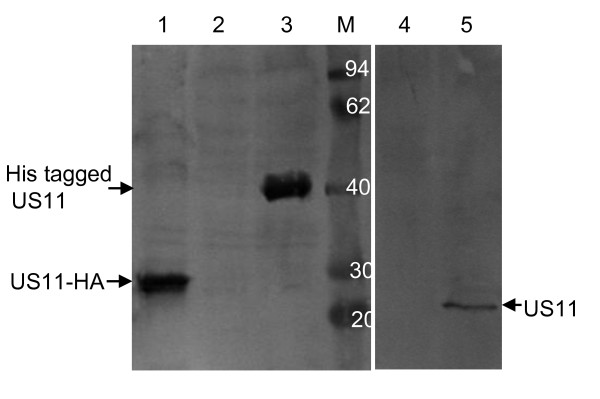
**Western blot analysis of US11 with anti-US11 polyclonal antibody**. Lane 1, the lysate of HEK293T cells transfected with plasmid encoding full-length US11-HA protein; Lane 2, total protein from *E. coli *BL21 (DE3); Lane 3, total protein from *E. coli *BL21 (DE3) harboring the plasmid pET-32a-US11 after induction by IPTG; Lane 4, the lysate of mock infected Vero cells; Lane 5, the lysate of Vero cells infected with HSV-1; Lane M, the protein maker (kDa). Arrowheads indicate the position of the US11 protein.

After three immunizations, the rabbit antiserum was collected as previously described [16]. Western blot results demonstrated that the anti-US11 polyclonal antibody reacted with one band with apparent molecular masses of 21 kDa in Vero cells infected with HSV-1 (Figure 3, lane 5) at a dilution of 1:800. In contrast, no band was detected in mock infected Vero cells (Figure 3, lane 4). Moreover, the anti-US11 polyclonal antibody could recognize the band with apparent molecular weight of 40 kDa in lysate of *E. coli *BL21 (DE3) transformed with the plasmid pET-32a-US11 after induction by IPTG (Figure 3, lane 3) and the expected 23 kDa band in lysate of HEK293T cells transfected with plasmid encoding US11-HA protein (Figure 3, lane 1), whereas no band was detected in lysate of *E. coli *BL21 (DE3) (Figure 3, lane 2).

**Figure 4:**
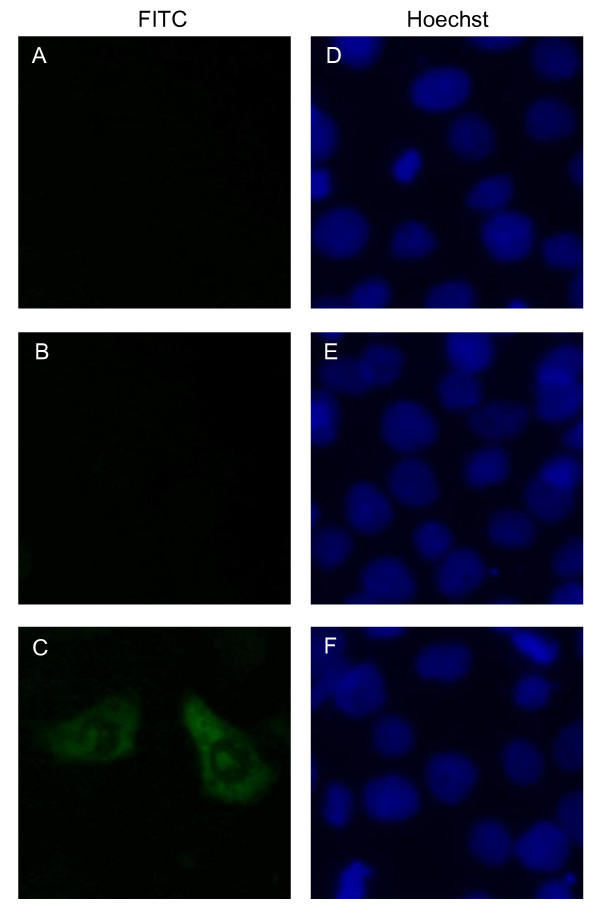
**Indirect immunofluorescent assay of the US11 protein in Vero cells infected with HSV-1**. (A) HSV-1 infected Vero cells probed with the preimmune serum. (B) Mock infected and (C) HSV-1 infected Vero cells probed with the US11 antiserum. Cells were labeled with FITC-conjugated goat anti-rabbit immunoglobulin G (A-C) and counterstained with Hoechst to visualize the nuclei (D-F). Magnification, 40×.

Immunofluorescent assay revealed that anti-US11 polyclonal antibody identified the US11 protein expressed in the cells infected with HSV-1. The results demonstrated that the US11 protein localized in the cytoplasm and nucleolus of the infected cell (Figure 4C), which is consistent with previous reports [9,17]. In contrast, there was no staining in mock infected cells (Figure 4B) or HSV-1 infected cells detected with the preimmune serum (Figure 4A). These results suggested that the polyclonal antibody had good reactivity and specificity against the US11 protein in infected cells, which were consistent with previous studies involved [10-12,18].

## Discussion

In general, recombinant proteins are the most common source of the diagnostic reagents and can be expressed in mammalian, insect and bacterial cells [19-21]. While each of these systems has its advantages [22], the bacterial expression system is one of the most universally used and has been employed widely due to multiple factors, including its relative inexpensive cost, ease of manipulation and rapid growth rate [23]. However, different codon usage pattern in *E. coli *can cause diminished the production of heterologous eukaryotic protein. Many *E. coli *strains, such as BL21 CodonPlus and Rosetta-2 derived from BL21, are optimized to enhance expression of gene sequences that contain codons used rarely by *E. coli *[24]. Because there are a few rare codons in the US11 gene, three different *E. coli *strains, including *E. coli *BL21 (DE3), BL21 CodonPlus and BL21 Rosetta were used to optimize the expression of the fusion protein. As a result, the His-tagged US11 protein could be expressed in all of these three bacteria hosts, but with a slight better expression in *E. coli *BL21 (DE3) (data not shown). Additionally, different expression parameters were tested to optimize the expression of the US11 protein, demonstrating that the US11 protein gained the highest expression under the condition of 37°C (Figure 2A, lane 7) with 1 mM IPTG (Figure 2A, lane 3) for 5 h (Figure 2B, lane 6).

It is very common that high level expression of recombinant proteins in *E. coli *result in the formation of insoluble and inactive aggregate known as inclusion bodies [25]. Although the proteins in the inclusion bodies are easy to purify and are protected from the intracellular protease, they are synthesized in the form of misfolding or partial folding polypeptides. Therefore, purified inclusion bodies must undergo the process of protein renaturation. In the present study, denaturation solution was removed by dilution or dialysis, which permitted renaturation of the His-tagged US11 proteins. However, the proteins purified from the inclusion bodies are not suitable for the researches that require the correct protein conformation.

The US11 protein is among the most-abundant viral proteins present in cells late in infection and is packaged in the tegument of the native virions [17]. In this study, anti-US11 polyclonal antibody reacted with one band with apparent molecular masses of 21 kDa in Vero cells infected with HSV-1 for 24 h., which is consistent with previous reports used by monoclonal or polyclonal antibodies against US11 [4,10-12]. The specificity of our US11 polyclonal antibody was confirmed by both WB and IFA in transfected and infected cells, and it also worked well, which maybe special advantages of our antibody over those antibodies. The subcellular localization of a viral protein determines its function. It is reported that soon after HSV-1 infection, the US11 protein is found in the cytoplasm, either as heterogeneous oligomers or associated with ribosomes or both [26,27]. Later during infection, the US11 protein accumulates into nucleoli and is also found in RNP fibrils as well as in clusters of interchromatin granules. As expected, our results also demonstrate that the US11 protein localizes in the cytoplasm and nucleolus of the infected cell.

Early studies have demonstrated US11 is a multifunctional protein involved in posttranscriptional regulation of gene expression and in biological processes related to the survival of cells following environmental stress [11]. To further gain insight into the molecular mechanisms underlying the multiple functions of this protein, identification of cellular factors capable of interacting with the US11 protein is necessary. Therefore, the anti-US11 polyclonal antibody may serve as a useful tool for further study of US11 interaction partners by co-immunoprecipitation.

## Conclusions

In the present study, we obtained a high-level expression of the recombinant US11 protein as well as high titers of rabbit polyclonal antibody against US11 protein specially. The anti-US11 serum was able to detect the US11 protein in HSV-1 infected cells and the US11 protein localized in the cytoplasm and nucleolus of the infected cell. This special polyclonal antibody provides a good tool for further studying structural and functional characterization of HSV-1 US11 protein.

## Materials and methods

### Construction of the plasmid expressing the His-tagged US11 protein

The US11 gene of HSV-1 (strain F) was amplified by PCR using KOD plus polymerase (Toyobo, Osaka, Japan) from the pYEbac102 [28] using the following primers: 5' CGGAATTCATGAGCCAGACCCAACC 3' and 5' ACGCGTCGACTACAGACCCGCGAGCCGT 3' as previously described. The purified PCR product was digested with *Eco*RI and *Sal*I (Takara, Dalian, China), and inserted into pET-32a (+) (Novagen, Shanghai, China) to generate a recombinant plasmid pET-32a-US11.

### Expression of the His-tagged US11 protein

*E.coli *BL21 (DE3) was transformed with pET-32a-US11 and the transformant was inoculated into LB medium containing 50 μg/ml ampicillin and grown for 12 h at 37°C. The culture was transferred to fresh LB medium and grown at 37°C until the OD_600 _reached about 0.5. Then the recombinant protein was induced by isopropyl-β-D-thiogalactopyranoside (IPTG) at a final concentration of 1 mM for 4 h. The total bacterial lysates were analyzed by SDS-PAGE [29].

To increase the yield, the expression of the His-tagged US11 protein was optimized as previously described [16] at different temperatures (30°C and 37°C), different concentrations of IPTG (0.5, 1.0, 1.5 and 2.0 mM), and varying durations of induction (0, 1, 2, 3, 4, 5 and 6 h). Protein expression was assessed by SDS-PAGE.

### Analysis of the solubility of the His-tagged US11 protein

The analysis was performed as previously described [16].

### Purification and renaturation of the His-tagged US11 protein

The purification and renaturation of the His-tagged US11 protein were performed as previously described [16]. After analysis by 12% SDS-PAGE, the yield of the purified His-tagged US11 protein was quantified by Coomassie (Bradford) Protein Assay Kit (Pierce, Beijing, China) [30].

### Production of polyclonal antibody against the His-tagged US11 protein

The immunizations were performed as previously described [16], except that the His-tagged US11 fusion protein was used in this study.

### Western blot

Western blot was performed as previously described [16], except that the anti-US11 polyclonal antibody (1:800 dilution) was applied in this study.

### Indirect immunofluorescent assay

Immunofluorescent assay was performed as previously described [16]. However, the Vero cells were infected with the HSV-1 at a MOI of 1 and fixed 20 h after infection. Additionally, the anti-US11 polyclonal antibody at a dilution of 1:800 was applied in this study.

## Competing interests

The authors declare that they have no competing interests.

## Authors' contributions

YH carried out most of the experiments and drafted the manuscript. W participated in construction of US11 plasmid. SY have critically revised the manuscript and the experimental design. All the authors read and approved the final manuscript.
